# Assessing the Antifungal Activity of a Soft Denture Liner Loaded with Titanium Oxide Nanoparticles (TiO_2_ NPs)

**DOI:** 10.3390/dj11040090

**Published:** 2023-03-29

**Authors:** Amal Qasim Ahmed, Sattar Jabbar Abdul-Zahra Al-Hmedat, Dunya Malhan Hanweet, Julfikar Haider

**Affiliations:** 1 Department of Prosthodontics, Faculty of Dentistry, University of Kufa, Najaf 54001, Iraq; 2 Department of Conservative Dentistry, Faculty of Dentistry, University of Kufa, Najaf 54001, Iraq; 3 Department of Oral Pathology, Faculty of Dentistry, University of Kufa, Najaf 54001, Iraq; 4 Department of Engineering, Manchester Metropolitan University, Manchester M1 5GD, UK

**Keywords:** antifungal agent, titanium oxide, nanoparticles, denture liners, polyethyl methacrylate, *Candida albicans*

## Abstract

Aim: Soft denture lining materials are susceptible to be colonized by different microorganisms, especially by *Candida albicans* (*C. albicans*), causing denture-induced stomatitis. This study was designed to evaluate the effectiveness of incorporating titanium dioxide nanoparticles (TiO_2_ NPs) into a soft denture liner towards reducing microbial activity. Method: A total of 40 PEMA-TiO_2_ nanocomposites samples were fabricated by adding 0.0 wt.% (control), 1.0 wt.%, 1.5 wt.%, and 2 wt.% TiO_2_ NPs to a heat cured soft denture lining material (polyethyl methacrylate, PEMA). The prepared samples were divided into four groups (*n* = 10) according to the content of TiO_2_ NPs. The uniformity of TiO_2_ NPS distribution within the denture liner matrix was assessed using a Scanning Electron Microscope (SEM). The viable count of *C. albicans* was evaluated to test the antifungal resistance of the developed composite. Results: The SEM images showed fairly homogeneous dispersion, with patches of TiO_2_ NPs agglomeration within the PEMA matrix and an increasing concentration of NPs with higher NP content. The particle map and EDX analysis confirmed the evidence of the TiO_2_ NPs. The mean viable count results for the control (0.0 wt.%) and 1.0 wt.%, 1.5 wt.%, and 2 wt.% TiO_2_ groups were 139.80, 12.00, 6.20, and 1.00, respectively, with a significant difference from the control group (*p* < 0.05). The antifungal activity also increased with the increase in the concentration of TiO_2_ NPs. Conclusions: The addition of TiO_2_ NPs into a heat-cured soft denture liner provided antifungal activity as evidenced by the reduced colonization of *C. albicans.* The antimicrobial activity of the liner material increased with the increased concentration of TiO_2_ NPS.

## 1. Introduction

When a removable denture base has good adaptation to the underlying oral supporting tissues, the denture’s fitting, stability, retention, and occlusion, as well as the patient’s appearance, are improved. However, over time, these underlying supporting tissues resorb, leading to instabilities in adaptation and in the patient’s appearance and comfort [1]. Poorly fitted dentures can adversely affect the underlying thin oral mucosa, forcing some patients to be unable to wear the unstable and hard removable dentures, which can cause irritation to the fragile and thin mucosa [2,3]. In order to readapt the denture base to its supporting tissue, denture relining should be utilized. The application of a soft liner material to the surface of the denture can reduce tissue irritation due to its resiliency and thus the patients would have a more comfortable denture wearing experience, without any pain [4,5].

The liners absorb and redistribute the applied forces of mastication by providing a layer of cushion, and thus the irritated and inflamed fragile oral tissues can restore their integrity and health, enhancing patient’s masticatory ability, chewing efficiency, and biting forces by improving the removable denture’s fitting and retention [5,6]. Despite the advantages found by relieving the oral irritated mucosa, the soft liners possess many shortcomings, which can be identified clinically when patients wear their relined dentures [7]. 

The most important issue is the invasion of such materials by different pathological microorganisms (predominantly *C. albicans* fungi), which colonize, attach, and disrupt the material integrity, causing oral mucosal infections called denture stomatitis. This makes denture wearing a more painful and challenging for the patients [8]. Denture stomatitis is a frequent pathological condition with redness and a burning sensation of underlying denture supporting mucosa (with both complete and partial dentures), and this more common in maxillary ridges than in mandibular ridges [4,9]. The term Candida comes from the Latin word candid, which literally means white. In general, Candida is a Gram positive fungus, with an oval or round shaped cell with a thin wall and very small diameter ranging from 3 to 30 μm. Candida have many different species, but the most predominant one is *C. albicans,* which represent more than 80% of the species isolated from the oral cavity [10,11]. 

The denture base, with its soft liner, should be cleansed thoroughly to avoid such colonization by *C. albicans,* and hence denture-related stomatitis. Many chemical and mechanical methods are used to disinfect the denture from the attached fungi, but excessive use of these disinfectants will adversely affect the serviceability of both materials [12]. However, using of these conventional cleansing methods might be compromised in some handicapped, geriatric, and hospitalized patients because of their difficulties with movement, loss of memory, and perceptive impairment [13].

Many research studies had been conducted and are still continuing in order to obtain a suitable solution for the problem of *C. albicans* colonization. Al-Shammari et al. demonstrated that zein NPs could be a potential candidate as an antifungal agent, and their effectiveness can be expanded by loading them with transitional metal ions such as copper or chromium. Silver nanoparticles have also been shown as a potent inhibitors of *C. albicans* biofilm formation [14,15]. One research direction focused on adding antifungal agents within the soft liner material [16]. According to previous studies, TiO_2_ NPs are considered to be one of the most effective antifungal agents. Its antifungal or antimicrobial property is related to its crystal structure, shape, and size [17]. TiO_2_ NPs also exhibit eco-friendly biocidal properties, which are attributed to their strong oxidizing potential. It has been used against a wide range of infectious microbes, including various bacterial strains, endospores, fungi, algae, protozoa, viruses, microbial toxins, and prions [18]. TiO_2_ NPs are used for treating skin infections and by reducing the size of the nanoparticles, its antimicrobial activity can be increased [12,19]. In dentistry, the addition of TiO_2_ NPs is preferred because of their, excellent biocompatibility, low cost, high refractive index, white color, and corrosion resistance, as well as their chemically inactive, nontoxic, and excellent mechanical properties. Titanium is considered one of the fourth most available metals on Earth, following aluminum, iron, and magnesium [20,21,22].

Although TiO_2_ NPs have been found to be effective in dental applications as an antimicrobial agent in denture base materials, the antifungal efficacy of TiO_2_ NPs when added to a soft denture liner, such as being heat cured polyethyl methacrylate (PEMA), is lacking. Furthermore, the response of TiO_2_ NPs to *Candida albicans* for this kind of application has not yet been explored. Therefore, the current study will bring a novel understanding for assessing the efficacy of TiO_2_ NPs into the PEMA soft denture liner. The null hypotheses were as follows:

1. The addition TiO_2_ NPs in the PEMA denture liner would not make any difference in the viable count of *C. albicans*.
2. With increasing the concentration of TiO_2_ NPs in PEMA, the viable count of *C. albicans* would not be affected.

## 2. Materials and Methods

### 2.1. Materials

The materials used in this study for sample preparation and conducting antifungal tests are presented in Table 1.

### 2.2. Fabrication of Soft Liner Samples

#### 2.2.1. Mold Preparation

**Figure 1:**
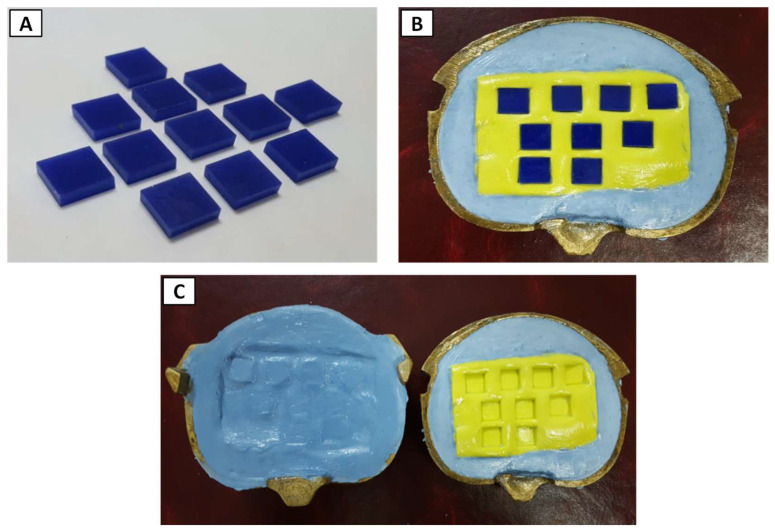
(**A**) Plastic patterns and (**B**) plastic patterns imbedded in silicone mold and stone, and (**C**) the molds after removing the patterns.

Plastic patterns with a 10 mm × 10 mm square cross section and 2.3 mm thickness (Figure 1A) [23] were used to prepare the specimens, which were first placed in the silicone duplicating material and then allowed the silicone to be set. The silicone mold with its impeded plastic patterns was placed in a dental stone mixture that was prepared following the manufacturer’s instructions with a water to powder ratio of 25 mL/100 g, which was then poured in the lower part of a metal dental flask (Figure 1B).

After setting of poured stone in the lower part, a coating of a separating medium was applied to the stone and plastic patterns. The two halves of the flask were assembled by placing the upper part on the lower part and then loaded with dental stone again and allowed extra time for complete stone setting. The flask was opened, and the plastic patterns were taken out from the duplicating silicone, leaving their places in it, which acted as a mold (Figure 1C).

#### 2.2.2. Preparing Heat Cure PEMA Soft Liner

As stated in the manufacturer’s instructions, the P/L (soft liner powder/liquid monomer) ratio for each 1 mL of liquid monomer to 1.2 g of powder was considered. In a dry small and clean glass bowel, the liquid was mixed with the powder and left until it reached the stage in which it could be used (dough stage). The insulating material was placed on both halves of the flask and left to dry in order to apply the mixed acrylic- based soft liner.

#### 2.2.3. Incorporation of Titanium Nanoparticles

In three different concentrations (1%, 1.5%, and 2% by weight), TiO_2_ NPs were added into the liner monomer for making the experimental specimens. The concentrations were selected based on a pilot study and values reported in the literature. The mixture was scattered for 3 min to disperse in into separate nanocrystals by utilizing probe sonication apparatus at 120 W and 60 KHz. The bowel containing suspension of the monomer and TiO_2_ NPs was placed in an ice-water bath to cool by eliminating the generated heat of sonication, which might lead to changes in the suspension volume or cause degradation. 

The formed monomer suspension with TiO_2_ NPs was immediately added and mixed with the PEMA powder to avoid any particle agglomeration. The proportioning and mixing of the loaded soft liner with these nanoparticles were carried out in the same way as previously mentioned for the control specimens. In order to maintain a precise P/L ratio, the weight of the TiO_2_ NPs powder was subtracted from the weight of the PEMA powder.

**Figure 2:**
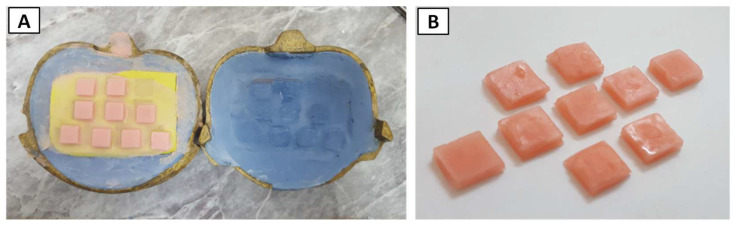
Soft liner specimens after curing (**A**) within the molds and (**B**) outside the molds.

When the soft liner reached the dough stage, it was packed into the mold and the curing process was accomplished in a thermostatically controlled water bath in two steps, as suggested by the manufacturer. In the first step, the flask containing the samples was heated up to a temperature of 70 °C and maintained the temperature for 90 min, while in the second step, the temperature was raised up to 100 °C and maintained for 30 min. Again, the cooling process of the flask was conducted at room temperature for 30 min, followed by complete cooling with tap water for 15 min before deflasking. The specimens were taken out from the flask (Figure 2) and a sharp blade was used to remove any excessive material attached to the samples, and fine grit silicon polishing bur (Vertex) and fine grit sand paper were employed to further remove any sample flash. Finally, sterilization of the specimens was conducted in an autoclave.

### 2.3. Isolation of Candida albicans

*C. albicans* were isolated from the oral cavities of 25 patients who attended the clinic of oral medicine of dentistry College, University of Kufa, Iraq, with a complaint of denture stomatitis with oral thrush. The process of isolation was conducted using a sterile cotton swab, which was gently scrubbed over the lesional site. Sabouraud dextrose agar (SDA) was used as the primary isolation medium for the subsequent inoculation [24]. The collected swabs were cultured and incubated aerobically at 37 °C for 1–2 days followed by preservation at 4 °C for later investigation.

### 2.4. Identification of Candida albicans

The samples taken from the oral cavity have more than one species of candida; hence, it is important to differentiate between the different species. Several methods were used to identify and confirm that the collected fungi were *C. albicans*.

#### 2.4.1. Colony Morphology and Microscopical Observation

**Figure 3:**
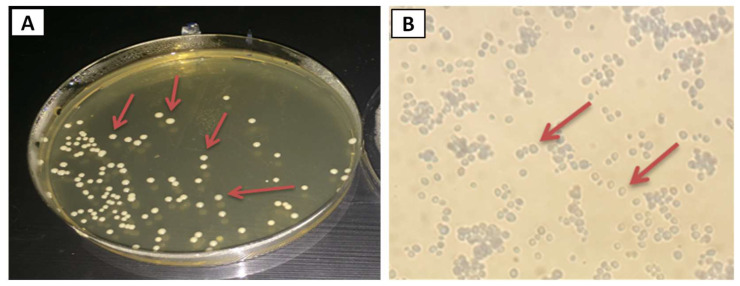
(**A**) Photographs of colonies of *C. albicans* on SDA and (**B**) optical microscopy image of candida, which appears as Gram-positive small oval or budding yeast cells.

When a suitable medium and conditions were provided for the growth of *C. albicans* fungi, clear, convex, creamy and smooth colonies formed on the SDA medium (Figure 3A).

By staining with Gram’s stain method, the most commonly used differential staining technique to differentiate between Gram-positive or Gram-negative microorganisms, *C. albicans* can be defined microscopically. First, from one isolated colony of candida, a little amount was mixed with a drop of normal saline on a glass slide, the formed suspension was distributed on this slide and was left to dry completely, and it was fixed by passing it over the flame of a Bunsen burner to be ready for staining with the basic dyes and materials of this method (crystal violet, gram’ iodine, acetone–ethanol, and safranin) [25]. Finally, the glass slide was examined under a light microscope to see the stained candida (Figure 3B).

Sabouraud dextrose broth was made ready based on the instructions provided by the manufacturer. First, 30 g of SDB powder was dissolved in one liter distilled water and sterilized in an autoclave at 121 °C and 103.42 kPa for 15 min. A controlled cooling procedure was followed to bring down the culture media temperature to 47 °C. It should be noted that to each 1 L of medium, 0.05 g of chloramphenicol antibiotic was added to avoid any bacterial growth. 

#### 2.4.2. Germ Tube Formation

**Figure 4:**
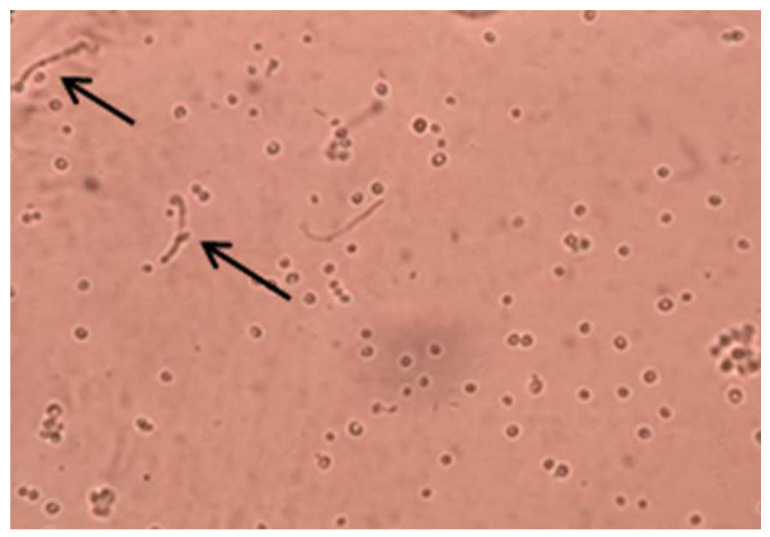
Formation of the *C. albicans* germ tube.

A tiny amount of fungal cells were collected from an isolated colony suspended in 0.5 mL of serum, and the tube was incubated at 35–37 °C for up to three hours. Under a low magnification, a glass slide containing a drop of fungi–serum suspension was investigated for the formation of germ tubes, as shown in Figure 4.

#### 2.4.3. Biochemical Identification

For the identification of the isolated fungus as *C. albicans*, the analytical profile index (API) Candida system (bioMérieux) was utilized. This system was composed of 10 various biochemical tests, which included dehydrated substrates in order to decide the usage profiles of the yeasts. The manufacturer’s instructions were followed to complete the identification procedure. A suspension made using 1–2 colonies of the isolated *C. albicans* was added to API NaCl 0.85% with turbidity equal to 3McFarland standard value. The tubules were loaded with yeast suspension. The tubules were closed by API-Set oil because of some of the reactions were anaerobic. The API strip was incubated at 37 °C for 18–24 h and the results were collected as per the API-Candida table. Identification was made by using a profile register provided by the maker, indicated as a four digit number.

### 2.5. Assessing Antifungal Activity of PEMA-TiO_2_ Nanocomposites

For assessing the antifungal functionality of the PEMA-TiO_2_ nanocomposites, *C. albicans* was diluted in a 0.9% NaCl solution. A McFerland densitometer was employed to prepare a yeast suspension of approximately 10^7^ colony forming units (CFU/mL) (0.5 McFarland standards). 

In a tube with a volume of 9.9 mL of Sabouraud dextrose broth, each sample was placed into the tubes with 100 μL of the yeast suspension and they were dispensed and incubated for one day at a temperature of 37 °C. This ensured a final concentration of cells with 10^5^ CFU/mL. The samples were gathered and washed five times following a standard method by immersing it in sterile deionized water to eliminate loosely joined cells. In Sabouraud dextrose agar plates, the viable cells attached to the sample surface were calculated [23,26] using Equation (1).

(1) $$AFE=\frac{V_{c}-V_{t}}{V_{t}}\times 100\%$$

where *AFE* is antifungal efficacy, and *V_c_* and *V_t_* are the number of viable fungal colonies in the control and experimental samples, respectively.

### 2.6. Characterization of Soft Liner Samples

Experimental and control samples were tested using a Fourier Transform Infrared Spectroscope (IR Prestige-21Shimadzu, Japan) to figure out whether there was a chemical reaction between the soft denture lining material and TiO_2_ NPs. A Scanning Electron Microscope (S50. FEI, The Netherlands) equipped with an Energy Dispersive X-Ray spectroscope (EDX) was used to examine both the control (pure PEMA) and experimental (PEMA-TiO_2_) samples, and to identify the distribution TiO_2_ NPs within the PEMA matrix. The samples were sputter coated with a thin layer of gold to make them conductive.

### 2.7. Statistical Analysis

Viable count results were statistically analyzed by SPSS software (version 21). Descriptive statistics such as the mean, standard deviation (S.D.), standard error (S.E.), and different quartile values were extracted from the raw data. Furthermore, the data were subjected to one-way analysis of variance (ANOVA) followed by a least significant difference (LSD) test to identify statistical significance with *p* < 0.05.

## 3. Results

### 3.1. Nano-Composite Characteristics

**Figure 5:**
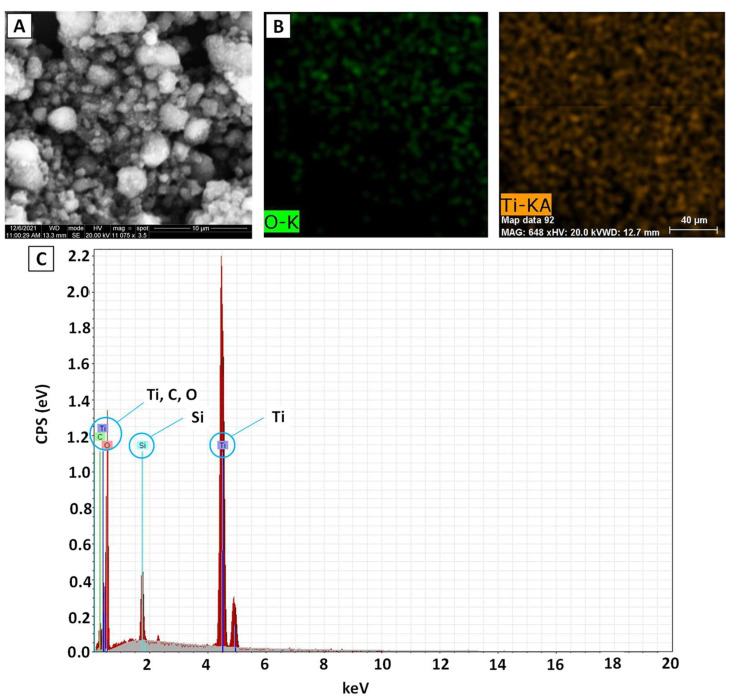
(**A**) SEM image, (**B**) Ti and O particle map, and (**C**) EDX analysis of the TiO_2_ NPs.

**Figure 6:**
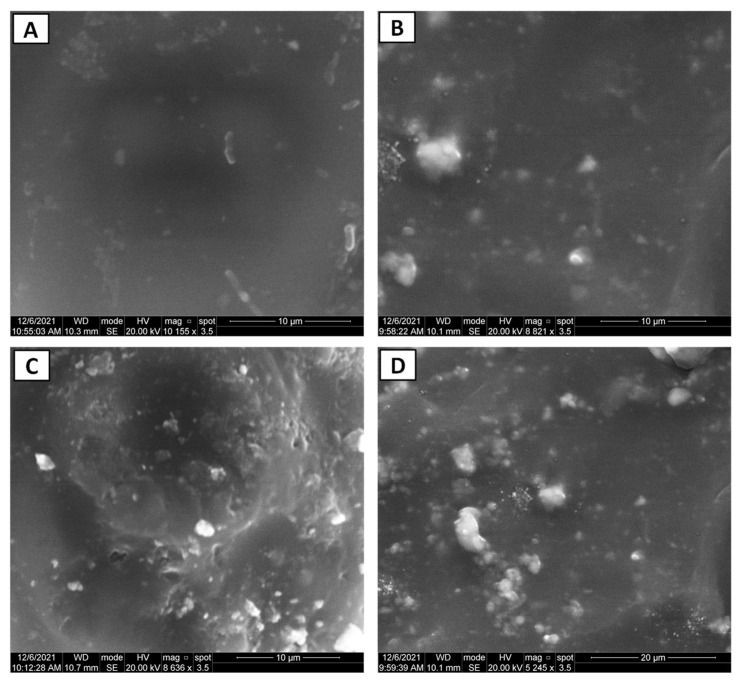
SEM images of (**A**) PEMA and PEMA-TiO_2_ nanocomposite: (**B**) 1.0 wt.% (**C**) 1.5%, and (**D**) 2.0 wt.%.

Figure 5A presents the morphology of TiO_2_ NPs. The particles were spherical in shape, but formed clusters. The particle map and EDX analysis showed evidence of the TiO_2_ NPs. In the authors’ previous investigation, it was established by FTIR spectroscopy that a similar shape of absorption peaks was found for both the experimental and control samples, and this indicated no chemical reaction between TiO_2_ NPs and PEMA [27]. Furthermore, the SEM images presented in Figure 6 demonstrate that TiO_2_ NPs were fairly uniformly distributed with some areas of aggregation within the PEMA matrix. A higher number of particles appeared along with the increase of TiO_2_ NPs concentration in the PEMA.

### 3.2. Evaluating Viable Count of C. albicans (CFU/mL)

**Figure 7:**
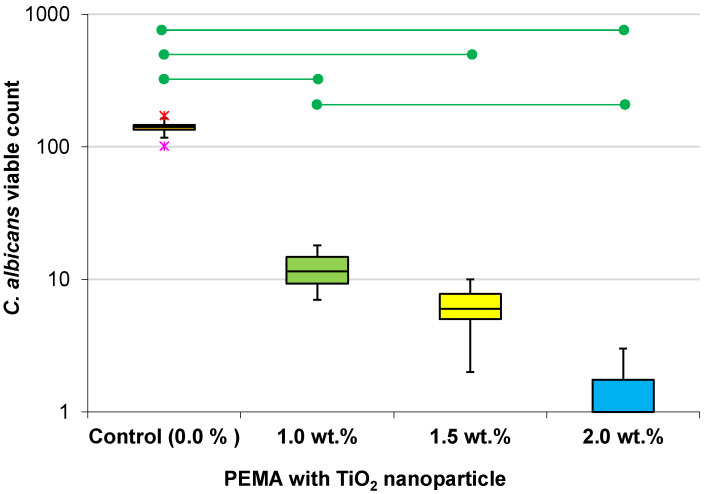
Box plot of the *C. albicans* viable count for different PEMA-TiO_2_ nanoparticle groups and the control group (pure PEMA). A horizontal line connecting two groups indicated a statistically significant difference.

Table 2 and Figure 7 present the results associated with the mean viable count of *C. albicans* and other descriptive statistics. All of the experimental groups showed progressively lower mean values with the increasing concentration of TiO_2_ NPs, with the lowest mean value of 1.0 for the 2 wt.% group compared with the control group mean count of 139.8. With the increase in TiO_2_ NPs concentration, AFE increased. With just 1.0 wt.% TiO_2_ NPs the AFE was over 90%. After a further increase in the nanoparticles by 0.5 wt.%, AFE increased by approximately another 4% and finally at 2.0 wt.% AFE increased to nearly 100%, indicating that the nanocomposite denture liner would be highly effective against *C. albicans*.

A one-way ANOVA of the fungal viable count results from the different experiments indicated that the difference between the groups was highly significant (Table 3).

Further statistical comparison was made using the least significant difference (LSD) test, where the control group was significantly different from each of the experimental groups, as shown in Table 4. However, no statistical significance was observed between 1.0 wt.% and 1.5 wt.% or 1.5 wt.%, and 2.0 wt.%.

**Figure 8:**
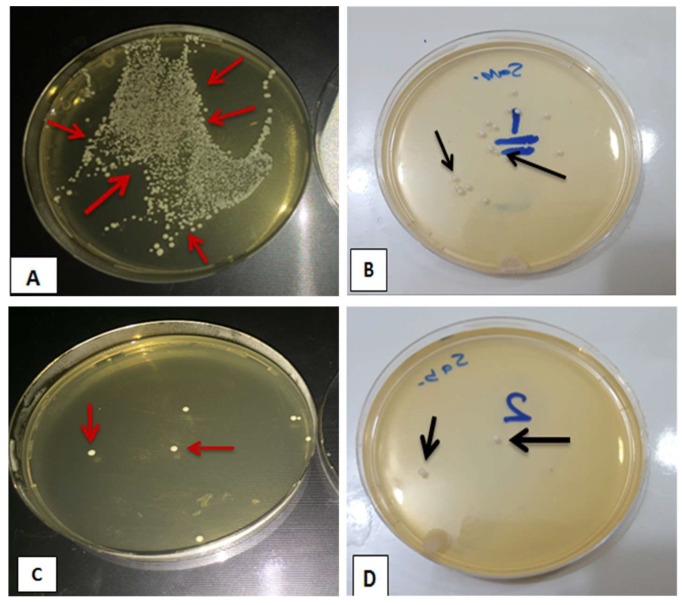
SDA plates showing CFU/mL of *C. albicans* for each group of study: (**A**) control with a large number of fungal colonies compared with the nanocomposites with different TiO_2_ NPs concentrations showing a decreased number of colonies: (**B**) 1.0 wt.% (**C**) 1.5%, and (**D**) 2.0 wt.%.

Figure 8 shows photographs of SDA plates showing the colony formation of *C. albicans*. The reduction in colonies were clearly visible with the increase in TiO_2_ NP concentration.

## 4. Discussion

In the next two decades, it is expected there will be an increase in the number of edentulous patients and thus a rising demand for wearing removable dentures. This would subsequently increase the need of using soft denture lining material to make the denture wearing a more comfortable experience for the patients [28,29,30].

Denture-related stomatitis caused by the colonization of *C. albicans* on the liners is considered to be one of the most significant problems. Therefore, the addition of nanoparticles such as TiO_2_ within the liner material itself might solve the problem to a greater extent, because this type of nanoparticle possesses a general antimicrobial activity and especially antifungal activity [17,31]. In this study, an attempt was made to develop a nanocomposite by adding TiO_2_ NPs to the PEMA soft denture liner for resisting any microbial growth, especially against *C. albicans*, which would help to avoid a sore mouth from dentures.

From the statistical analysis of the viable count results obtained in this study, a highly substantial reduction in the CFU/mL of the *C. albicans* was found with the addition of TiO_2_ NPs into the PEMA liner material, and the antifungal activity of PEMA was increased with the increased concentration of the TiO_2_ nano material. Even with 1.0 wt.% TiO_2_ addition, an almost 10-fold decrease in *C. albicans* colony formation was achieved. Therefore, both of the null hypotheses were rejected.

TiO_2_ NPs exhibit eco-friendly biocidal properties, which is attributed to their strong oxidation potential. It has been reported that an electromagnetic attraction exists between the negatively charged surface of the microbe and the positively charged metal oxide nanoparticles, and because of this affinity, the death of the microbe will occur from the toxicity released by the metal oxide nanoparticles [32,33,34]. Another possible explanation is that TiO_2_ NPs can destroy *C. albicans* cells through the production of intracellular reactive oxygen species (ROS), which have undesirable effects inside the yeast cells with Coenzyme A oxidation and lipid peroxidation, leading to reduced adhesion and distorted ionic balance, causing deficient respiratory activity, which means inhibiting the respiratory cytosolic enzymes and modifying the macromolecule structures and eventual cell death [35]. In addition, cell destruction might happen because of the rupture in the cell membrane of *C. albicans*. TiO_2_ NPs can disturb *C. albicans* integrity causing loss of intracellular ions and other important substances. Furthermore, defecting the natural cell cycle at the pre-mitotic phase (G2/M) could lead to suppression of the budding process [18,36].

TiO_2_ NPs possess a large active surface area, which allowing for antifungal activity at a small dose of 1.0 wt.% so as to prevent *C. albicans* attaching, spreading, colonizing, and forming biofilms on the soft liners. Therefore, direct incorporation TiO_2_ NPs in the acrylic soft liner could create a sustainable antifungal effect. However, special attention should be given to the fact that the addition of nanoparticles must not significantly affect other physical, mechanical, optical, or aesthetic properties. In a previous study, it was found that adding 2.0 wt.% TiO_2_ NPs to PEMA soft liners resulted in a decrease in hardness, no change in shear bond strength, and an increase in the opacity of the liner material [27]. Similar results regarding the degradation of the mechanical and color properties of the PMMA denture base with the addition of TiO_2_ NPs were also reported [37,38,39]. It was found that the surface hardness of a nano-filler reinforced PMMA increased when the TiO_2_ NPs concentration was increased [37]. The authors attributed these findings to an improved chemical bonding between the PMMA matrix and the nano-fillers and a uniform distribution of the TiO_2_ NPs within the matrix facilitated by the coating of a silane coupling agent on the fillers.

No direct studies were found in the literature to compare the effect of TiO_2_ NPs on resisting fungal colonization in denture liners. However, studies have been reported on the antimicrobial resistance of NPs in PMMA denture base materials. For instance, this study was in agreement with Haghighi et al. [35], who investigated the antifungal activity of TiO_2_ NPs on the fungal biofilms that consisted of fluconazole resistant standard strains of *C. albicans*. Their study showed an enhanced antifungal activity of TiO_2_ NPs against the biofilms. Akhtar et al. (2016) [31] and Durairaj et al. (2015) [32] showed that TiO_2_ NPs had a super antifungal effect against Trichoderma reesei, Microsporum canis, Aspergillus niger, Penicillium sp, and Rhizopus. Totu et al. [40] reported that by incorporating only 0.4% TiO_2_ NPs in the PMMA polymer matrix with a stereolitographic technique could bring significant antibacterial effects to Candida species. 

Waly [41] studied the influence of adding 5.0 wt.% pure titanium dioxide (TiO_2_) and silver-doped titanium dioxide (Ag-doped TiO_2_) nanoparticles in one soft acrylic liner material on the antifungal activity. It was concluded that by incorporating Ag-doped TiO_2_ NPs into the acrylic liner produced an antifungal activity against *C. albicans* when exposed to visible light. Although TiO_2_ NPs did not affect the viscoelastic properties of the soft liner but the Ag-doped TiO_2_ NPs displayed a reduced cushioning effect within the clinically acceptable range. 

A clinical study was carried out to assess the efficacy of adding silicon dioxide (SiO_2_), zirconium oxide (ZrO_2_), and titanium oxide (TiO_2_) nanoparticles in a cold-cured Acrostone soft liner on *C. albicans* (CA) [42]. For each metal oxide nanoparticle, the concentrations were varied as 0.0 wt.%, 3.0%, and 7.0 wt.% in maxillary complete dentures, and swabs were collected from the relining sites after 14 and 28 days. The results suggested that CA adhesion increased with the increasing concentration of nanoparticles, and the antimicrobial resistance from highest to lowest order was as follows: TiO_2_ > ZrO_2_ > SiO_2_. However, 7.0 wt.% was recorded to provide the best efficacy in contrast with this study, where even 2.0 wt.% TiO_2_ NP showed the best antifungal activity. This could be due to the difference in the soft liner material. Another recent study identified a good inhibitory effect against the growth of *C. albicans* using ZrO_2_ NPs in a heat-cured acrylic-based soft lining material at concentrations of 1.5 and 2.0 wt.%. This could provide antifungal characteristics, reducing the chances of inducing denture-induced stomatitis. Furthermore, the shear bond strength also increased significantly with 2.0 wt.% ZrO_2_ NPs [43].

The antimicrobial efficacy of TiO_2_ could be credited to the hydrophilic nature and oxidative activity of TiO_2_. Upon adding TiO_2_ NPs to the denture liner, the same hydrophilic nature was transferred to the resulting nanocomposite, inhibiting the growth of microorganisms and preventing the formation of denture plaque [44,45]. Al Qahtani et al. introduced a new technique of denture base layering in order to incorporate TiO_2_ NPs in PMMA and to evaluate its effectiveness on *C. albicans* adhesion. Furthermore, surface roughness, hardness, translucency, and flexural strength characteristics were also quantified. The PMMA-TiO_2_ nanocomposites developed by the layering technique were shown to be effective in the reduction of *C. albicans* adhesion, while the surface and mechanical properties were unaffected [46]. Other than adding TiO_2_ NP as a filler in the denture base or denture liner acrylic, TiO_2_ was applied as a coating on the acrylic resin and its antimicrobial efficacy was assessed. It was found that the coating could effectively resist the growth of *C. albicans*. The major advantage of the coating is that it does not alter the mechanical properties of the acrylic material [47,48]. 

Although encouraging results on the antifungal resistance of PEMA-TiO_2_ nanocomposites were found in this study, further research needs to be carried out with other denture liner materials, such as cold cure, light cure, and silicone-based materials. Other mechanical (flexural or tear strength) and physical (color stability, sorption, and solubility) properties need to be assessed to ensure that the nanocomposite maintains its antifungal characteristics without compromising its durability. The results of this study cannot be directly applied to the clinical situation due to its in vitro nature. Most importantly, in vivo studies are required to demonstrate that the denture liner nanocomposite is effective for providing a long-term antifungal resistance.

Therefore, a low concentration of TiO_2_ NPs can be incorporated in the PEMA denture liner in every day clinical practice to enhance the antifungal activity in the oral cavity and to treat denture stomatitis with improved patient’s satisfaction.

## 5. Conclusions

Considering the shortcomings of this in vivo study, the key conclusions derived from this study are as follows.

(1) Microstructural analyses demonstrated uniform dispersion of TiO_2_ NPs in the PEMA polymeric matrix.
(2) Antifungal properties can be embedded within the soft denture lining material with the addition of TiO_2_ NPs into PEMA.
(3) The antifungal efficacy was found to be concentration dependent, with an increased antifungal activity when there was an increase in the concentration of TiO_2_ NPs.
(4) A concentration ranging from 1.0 to 2.0 wt.% TiO_2_ NPs in the PEMA denture liner could significantly reduce *C. albicans* colony formation in the oral environment compared with pure PEMA.

## Figures and Tables

**Table 1:** Details of the main materials used in this study.

Number	Description	Manufacturer
1	Vertex-soft Polyethyl methacrylate denture liner	Vertex, The Netherlands
2	Titanium dioxide nanoparticles (TiO_2_ NPs, anatase structure, 10–30 nm)	SkySpring, USA
3	Addition Silicone impression material	Vertex, The Netherlands
4	Type IV Dental stone	Zhermack, Italy
5	Sabouraud dextrose agar and Sabouraud dextrose broth	Oxoid, UK
6	API-Candida	BioMérieux, France

**Table 2:** Descriptive statistics and AFE of the *C. albicans* viable count results for the experimental and control groups.

Sample Groups	Mean	Standard Deviation	Standard Error	95% Confidence Interval for Mean		Min.	Max.	AFE (%)
				Lower Bound	Upper Bound			
Control (0.0 wt.%)	139.80	18.546	5.865	126.53	153.07	101	172	0.0
1.0 wt.%	12.00	3.916	1.238	9.20	14.80	7	18	91.42
1.5 wt.%	6.20	2.394	0.757	4.49	7.91	2	10	95.57
2 wt.%	1.00	1.054	0.333	0.25	1.75	0	3	99.28

**Table 3:** One-way ANOVA analysis of the *C. albicans* viable count.

	Sum of Squares	df	Mean Square	F	Sig.
Between Groups	134,072.300	3	44,690.767	488.246	0.000
Within Groups	3295.200	36	91.533		
Total	137,367.500	39			

**Table 4:** Least significant difference (LSD) test applied on *in. albicans* viable count results of all the studied groups.

Samples		Mean Difference (I-J)	Sig.
Control	1.0 wt.%	127.800 *	0.000
	1.5 wt.%	133.600 *	0.000
	2.0 wt.%	138.800 *	0.000
1.0 wt.%	1.5 wt.%	5.800	0.184
	2.0 wt.%	11.000 *	0.014
1.5 wt.%	2.0 wt.%	5.200	0.232

Note: * indicates significant difference.

## Data Availability

The data presented in this study are available within the article.
